# Constitutive and Stress-Induced Psychomotor Cortical Responses to Compound K Supplementation

**DOI:** 10.3389/fnins.2020.00315

**Published:** 2020-04-08

**Authors:** Shawn D. Flanagan, Felix Proessl, Courtenay Dunn-Lewis, Maria C. Canino, Adam J. Sterczala, Chris Connaboy, William H. DuPont, Lydia K. Caldwell, William J. Kraemer

**Affiliations:** ^1^Department of Human Sciences, The Ohio State University, Columbus, OH, United States; ^2^Neuromuscular Research Laboratory, Department of Sports Medicine and Nutrition, University of Pittsburgh, Pittsburgh, PA, United States

**Keywords:** ginsenoside, cortical activity, EEG, event-related potentials, source localization, exercise, psychomotor performance, stress

## Abstract

Isolated ginsenoside metabolites such as Compound K (CK) are of increasing interest to consumer and clinical populations as safe and non-pharmacological means to enhance psychomotor performance constitutively and in response to physical or cognitive stress. Nevertheless, the influence of CK on behavioral performance and EEG measures of cortical activity in humans is undetermined. In this double-blinded, placebo-controlled, counterbalanced within-group study, dose-dependent responses to CK (placebo, 160 and 960 mg) were assessed after 2 weeks of supplementation in nineteen healthy men and women (age: 39.9 ± 7.9 year, height 170.2 ± 8.6 cm, weight 79.7 ± 11.9 kg). Performance on upper- and lower-body choice reaction tests (CRTs) was tested before and after intense lower-body anaerobic exercise. Treatment- and stress-related changes in brain activity were measured with high-density EEG based on event-related potentials, oscillations, and source activity. Upper- (−12.3 ± 3.5 ms, *p* = 0.002) and lower-body (−12.3 ± 4.9 ms, *p* = 0.021) response times improved after exercise, with no difference between treatments (upper: *p* = 0.354; lower: *p* = 0.926). Analysis of cortical activity in sensor and source space revealed global increases in cortical arousal after exercise. CK increased activity in cortical regions responsible for sustained attention and mitigated exercise-induced increases in arousal. Responses to exercise varied depending on task, but CK appeared to reduce sensory interference from lower-body exercise during an upper-body CRT and improve the general maintenance of task-relevant sensory processes.

## Introduction

Ginsenosides (*Panax ginseng* C.A. Meyer, Araliaceae) have been used for thousands of years to improve cognitive functions (Oh and Kim, 2016; Seo et al., 2016), sickness behaviors (e.g., anxiety, depression, and fatigue) (Dang et al., 2009; Oh et al., 2015a, b; Zhang et al., 2016), and neuropathologic conditions that involve oxidative stress and inflammation (Joh et al., 2011; Yu et al., 2012, 2014; Lee et al., 2013; Baek et al., 2016). As the primary active ingredients, saponins possess low bioavailability in their unmodified forms (Lee et al., 2009). Industrial techniques are thus used to mimic the actions of the intestinal microflora that produce bioactive metabolites (Hasegawa et al., 1996, 1997; Bae et al., 2000; Kim et al., 2000; Chi et al., 2005; Ko et al., 2007). One metabolite, CK [20-*O*-β-D-glucopyranosyl-20(S)-protopanaxadiol] is of particular interest as it is readily absorbed into circulation and demonstrates efficacy in animal models of neurological disease (Park et al., 2012; Seo et al., 2016; Jakaria et al., 2018; Zong et al., 2019). In addition, CK appears to exert direct effects on the brain (Lian et al., 2005, 2006; Bae et al., 2010; Lee et al., 2010; Wang et al., 2010), but there is little physiological evidence in humans.

As one of the World’s best-selling herbs (Bai et al., 2018), there is a need to assess the effects of ginsenosides on objective measures of cognition and brain function in healthy humans (Smith et al., 2014). The visual choice reaction test (CRT) is a well-established psychometric paradigm that provides objective measures of psychomotor function based on response times. The CRT also produces robust patterns of brain activity that can provide insight into task-specific attention, memory, and psychomotor functions. If CK exerts favorable effects on these aspects of cognition (Smith et al., 2014; Jakaria et al., 2018), improvements in response times and electrophysiological measures are likely. Nevertheless, the influence of CK on task-related brain activity is unknown.

An additional consideration is that the effects of CK may be largely stress-inducible (i.e., apoptogenic) rather than constitutive (i.e., ergogenic; for review, see Smith et al., 2014). As a potent physiological stressor, acute exercise increases arousal (Otieno et al., 2019) and modulates performance on various cognitive tests including the CRT (Arcelin et al., 1998; Chang et al., 2012; Fontes et al., 2015). We recently demonstrated dose-dependent reductions in perceived exertion and circulating cortisol in response to intense acute exercise after two weeks of CK supplementation (Flanagan et al., 2018). Despite providing preliminary evidence that CK might improve cognition by mitigating exercise-induced arousal, more direct evidence is needed.

Event-related potentials (ERPs) and oscillations provide objective measures of cognition and are sensitive to the effects of caffeine (Barry et al., 2005, 2007), acetaminophen (Jaswal et al., 2019), and other nutritional substances. Numerous ERPs produced during the CRT are responsive to changes in arousal, attentional resources, working memory, and psychomotor function (Hallett, 1994; Toma et al., 2002; Polich, 2007; Luck and Kappenman, 2013). Event-related oscillations can also provide information on changes in arousal, sustained attention and working memory, top-down attentional control, multi-sensory stimulation, and local sensory integration (Jensen et al., 2007; Klimesch et al., 2007; Klimesch, 2012; Guntekin et al., 2013; Guntekin and Basar, 2016; Holroyd and Umemoto, 2016). In addition, more recent source analysis techniques such as current density source reconstruction (CDR) can provide information to confirm or explore the spatial characteristics of electrophysiological activity.

The purpose of this study was to explore the dose-dependent effects of CK on CRT performance and task-related brain activity. The CRT was used to provide insight into the effects of CK on selective attention, memory, and psychomotor processes critical for performance and to examine stress-inducible cortical responses to intense lower-body exercise. Given evidence for task-specific cognitive responses to exercise (Arcelin et al., 1998; Brisswalter et al., 2002; Chang et al., 2012), to clarify whether the inducible effects of CK might reflect changes in task-specific brain activity in addition to exercise-induced arousal, performance and brain activity were examined during two otherwise identical CRTs that emphasized the same lower-body musculature as exercise or upper body muscles that were largely uninvolved.

## Materials and Methods

### Experimental Approach

The participants, supplementation regimen, and experimental approach are fully detailed elsewhere (Flanagan et al., 2018). A general overview with considerations specific to this study is provided here. A double-blind, placebo-controlled, counterbalanced within-group study design was used. After familiarization with all procedures during an initial visit, participants were randomized to three 14-day supplementation cycles with a high CK dose (960 mg/day), low CK dose (160 mg/day), or placebo (0 mg/day). At the end of each cycle, participants completed a test visit with electromagnetic measures of brain activity and intense lower-body anaerobic exercise. A minimum of one week was provided between cycles for washout.

Each test visit began at a standardized time in the morning with confirmation of: (1) supplementation compliance, (2) 8 hour fasted state, (3) 6−8 hour of sleep the night before, (4) 24 hour drug, alcohol, and analgesic abstinence, (5) 72 hour exercise abstention, and (6) day 1−8 of follicular phase (as applicable). A 24 hour diet log was recorded prior to the first session and returned by participants with any modifications to confirm replication before each subsequent visit. A urine sample was collected to ensure adequate hydration based on refractometry (USG ≤ 1.025), with 500 mL of water provided when necessary. Participants then consumed six capsules of the prescribed treatment.

After confirmation of quality EEG signals, upper- and lower-body CRTs were performed, followed by intense lower-body anaerobic exercise and post-exercise CRTs immediately after that. Exercise was administered using a leg press device (Plyo Press; Athletic Republic, Park City, UT, United States) with five sets of 12 repetitions at 70% of one-repetition maximum (obtained during familiarization visit) and 2 mins rest between sets. Upper- and lower-body CRTs were administered on a commercially available touchpad device (The Quick Board, Memphis, TN, United States). To minimize the influence of learning effects, participants practiced the CRTs three times per week during supplementation [six practice sessions per cycle (18 total)].

### Participants

Nineteen healthy women (*n* = 10, age: 38.7 ± 7.8 years, height: 163.8 ± 4.4 cm, weight: 76.0 ± 11.6 kg) and men (*n* = 9, age: 41.2 ± 9.7 years, height: 177.4 ± 5.3 cm, weight: 88.5 ± 5.0 kg) volunteered to participate in this study. Participants were considered to have normal sensory and cognitive status and provided written confirmation of eligibility based the following conditions: medical clearance for intense exercise; no consumption of any ginseng-containing products; no food or supplement allergies; no hypersensitivity to caffeine-containing products; no limiting musculoskeletal injuries; no current use of any hormonal, anti-inflammatory, cholesterol- or blood pressure-lowering medications; absence of diabetes, hypertension, or cardiovascular disease; no tobacco use; and alcohol consumption of less than three drinks per day or 18 drinks per week. Individuals also indicated regular recreational activity but no participation in any structured physical training program. Prior to enrollment, interested individuals provided written informed-consent to participate in the study, which was approved by The Ohio State University Biomedical Sciences Institutional Review Board (2014H0404).

### Supplementation

Compound K was administered in an oral liquid capsule form (GINST15^®^, ILHWA Co, LTD., South Korea). Three capsules were consumed each morning and evening. For low dose supplementation, an active 160 mg capsule was consumed with two placebo capsules in the morning, and three placebo capsules were consumed in the evening. Supplementation adherence was recorded in a written log.

### Choice Reaction Test

**FIGURE 1:**
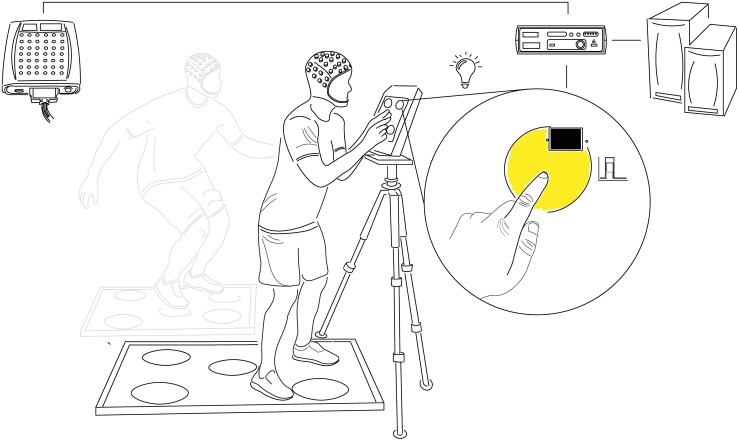
Choice Reaction Test Setup. Participants responded to the random illumination of one of five equidistantly spaced circles by touching the corresponding point on the presentation screen (upper-body CRT) or foot pad (lower-body CRT) as quickly and accurately as possible. Each test was comprised of 120 stimuli divided into three blocks of 40 with 30s rest between and a 500 ± 30 ms post-response stimulus onset delay. Stimulus onset and offset (response) timing were detected with photodiodes and marked at the corresponding timepoints in continuous EEG recordings with minimal delay or jitter (≤2 ms). Comparison of analog event markers and computer-generated response times confirmed the accuracy of stimulus and response co-registration. CRT, choice reaction test.

The CRT was administered on a 9.7^″^ tablet with a 120 Hz touch sample rate and 30 Hz frame rate (Apple Inc., Cupertino, CA, United States). During each CRT, visual stimuli (yellow circles) were presented randomly at one of five equidistant locations against a black background (Figure 1). Participants were instructed to respond as quickly and accurately as possible by touching the illuminated circle on the screen (upper-body) or the corresponding circle on a touch-sensitive board beneath them (lower-body). Each test consisted of three blocks of 40 trials (120 total stimuli) with 30 s rest between and a 500 ± 30 ms stimulus onset delay. For the upper-body CRT, participants were instructed to use the right or left index finger when stimuli were presented on the right or left side of the screen, respectively. When presented at the center position, participants were allowed to respond with either hand. For the lower-body CRT, participants were centered above the middle sensor and similarly instructed to respond with the left or right foot based on the location of stimuli presented on the tablet. Participants were also instructed to focus on the screen and avoid looking down at the board when responding.

The tablet was placed at a standardized relative height (eye-level) and distance for each participant (wrists parallel to tablet with arms fully extended). Stimulus contrast and intensity were consistent throughout the study. Stimulus and response timing were recorded with photodiodes placed on the outer edges of each presentation point using a stimulus tracking device (Stimtracker, Cedrus Corporation, San Pedro, CA, United States) and relayed to an EEG input module via a custom D-type cable. The accuracy of event marking was confirmed by comparing analog markers in the EEG recording to those generated by the CRT software.

### Electroencephalography

Brain activity was measured continuously during CRTs with high-density EEG using a 64-channel passive Ag-AgCl electrode-containing cap with ground and reference electrodes located at AFz and between Cz and CPz, respectively (expanded 10−20 configuration; Quik-Cap, Compumedics Neuroscan, Charlotte, NC, United States; No authors, 1994). Data were sampled from DC-2000Hz and amplified with 24-bit resolution, >10 GΩ input impedance, >110 db common mode rejection, and <0.5 uV peak to peak noise (SynAmps RT, Compumedics Neuroscan, Charlotte, NC, United States). After abrasion of scalp (comb) and electrode sites (blunted needle), the cap was placed in relation to the nasion–inion, preauricular, and vertex (Cz) landmarks, with locations recorded to maximize consistency and precision. Participants were instructed to wash and dry their hair the morning of each visit and avoid the use of any conditioners or other hair products. After the injection of electrode gel (Quik-gel, Compumedics Neuroscan, Charlotte, NC, United States), further abrasion and time were given as needed to achieve uniform impedances of ≤5 KΩ. Test blinks and jaw contractions were acquired and displayed to participants who were encouraged to avoid changes in gaze, unnecessary blinks, and jaw clenching during the CRTs.

#### Data Preprocessing

EEG data preprocessing and analysis were performed in a commercial software program (Curry 8, Compumedics Neuroscan, Charlotte, NC, United States). Digitized continuous EEG data were re-referenced off-line to a common average. When present (less than one random channel per participant on average), bad channels were interpolated with a distance-weighted average based on the nearest four channels. Continuous data were then filtered from 0.1−40.0 Hz (0.1 Hz HP w/2.0 Hz slope, 40.0 Hz LP w/5.0 Hz slope; Hann filter with 10% taper width). Artifact correction was applied on an individual basis. Artifacts from blinks and horizontal eye movements were removed with initial template matching using the VEOG and HEOG channels with amplitude and correlation thresholds set to maximize artifact waveform inclusion and minimize false positives. Artifacts were reduced based on visual comparison of averaged principal component (PCA) and artifact waveforms, with the minimum number of components removed (generally the first one or two) to reduce overcorrection. PCA was implemented with singular value decomposition using two rectangular data matrices (sensors and samples). When present, artifacts from electrocardiogram and electromyogram were removed with additional PCA analyses. Pre-processed samples were then segmented into epochs from −1000 to 1000 ms post-stimulus. Each epoch was manually inspected with any epoch containing excessive artifact marked for exclusion from further analysis. In addition, epochs containing any channels with signals greater (less) than 75 uV (−75 uV) were automatically excluded. Retained epochs were baseline corrected with channel-specific average subtraction based on the 500−0 ms pre-stimulus interval. The experimenter responsible for preprocessing was blinded to treatment condition. The number of trials used for analysis and SNR statistics are provided in Table 1.

**TABLE 1:** Summary of epoch statistics by treatment, segment, and timepoint.

**Treatment**	**Segment**	**Timepoint**	**Total (#)**	**Retained (#)**	**Retained (%)**	**Noise (uV)**	**SNR**
PLA	Upper	Pre	1713	1701	99.3%	0.418	2.84
		Post	1599	1557	97.4%	0.294	3.40
	Lower	Pre	1746	1341	76.8%	0.416	3.80
		Post	1671	1469	87.9%	0.369	3.34
160	Upper	Pre	1564	1554	99.4%	0.351	3.32
		Post	1577	1547	98.1%	0.326	2.87
	Lower	Pre	1627	1385	85.1%	0.455	3.30
		Post	1630	1403	86.1%	0.402	3.14
960	Upper	Pre	1721	1619	94.1%	0.371	3.70
		Post	1483	1456	98.2%	0.284	3.35
	Lower	Pre	1728	1372	79.4%	0.465	4.02
		Post	1608	1501	93.3%	0.369	3.19

*SNR, signal-to-noise ratio.*

### Data Analysis

Median response times were tabulated by segment (upper and lower), treatment (placebo, 160 and 960 mg), and timepoint (pre- and post-exercise) after the removal of lapses and false starts, which were defined as any value greater than two standard deviations above or below the individual mean response time.

**FIGURE 2:**
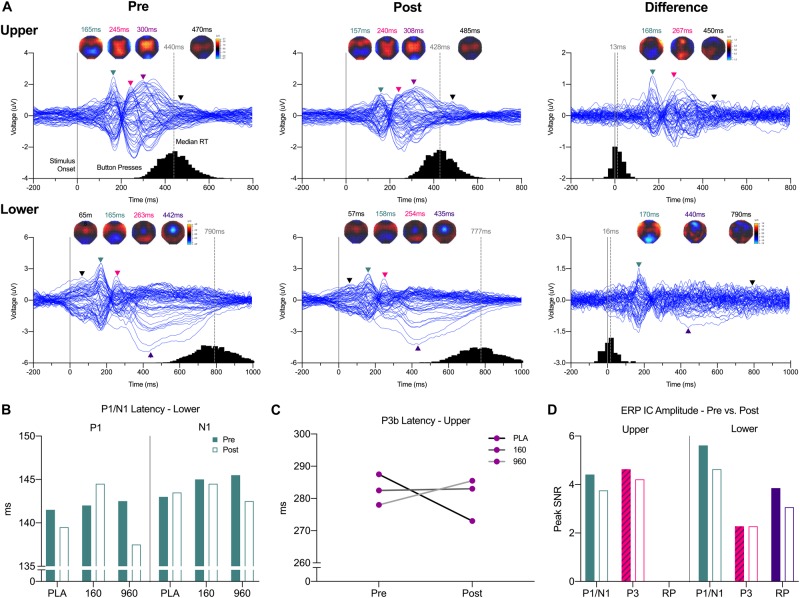
Event-Related Potential Waveforms, Response Times, and Interpolated Scalp Maps. **(A)** Grand-averaged 64-channel ERP tracings for each segment and timepoint plus the pre- minus post-exercise difference waveform (expanded 10–20 montage, common average reference). Waveforms are time-locked to stimulus presentation (solid gray line at 0 ms). For each trace, histograms of all qualifying response times are provided on the *X*-axis, with median response time indicted above the gray dashed lines. Black lines Triangles correspond to spherical spline interpolated 2D scalp voltage topography maps at the indicated time points with colors matched to identified ERP components and post-exercise map intensity scaled in relation to the corresponding pre-exercise condition. **(B)** Grand-averaged lower body P1/N1 latencies were reduced after exercise with 960mg, while a **(C)** dose-dependent increase in P3b latency after exercise was evident for the upper body. **(D)** As indicated by ICs for each respective ERP, amplitudes were generally lower after exercise. ERP, event-related potential; IC, independent component.

Initial examination of brain activity was performed on grand-averaged waveforms (GA WFs) for each segment, treatment and timepoint (Figure 2), with individual event-related potential (ERP) averages retained for statistical analysis. Preliminary analysis indicated no systematic differences between men and women, and the use of permutation statistics necessitated larger sample sizes than permitted when the analysis was divided by sex. Therefore, data from men and women were combined. The spatial and temporal properties of ERPs were explored with interpolated scalp topography maps of activity during the −200−700 ms (upper) and −200−1000 ms (lower) analysis intervals. Scalp topography maps were interpolated with spherical spline expansion. Peak locations common to each segment, treatment, and timepoint were used to select channels for component amplitude and latency measurements. Depending on component polarity, positive/negative area under the curve (AUC) and 30% fractional area latency (FAL) were computed (Luck, 2005). Windows for AUC and FAL were determined by: (1) aggregated visualization of GA WFs for the representative component channel across treatments and timepoints, (2) selection of onset and offset points to create a common analysis interval that encompassed the entirety of the respective component across conditions, (3) adjustment to create uniform signs specific to component polarity, (4) trapezoidal-based calculation of AUC (trapz() MATLAB function), (5) determination of minimum absolute sample value corresponding to 30% AUC, and (6) conversion of 30% AUC sample value to milliseconds from stimulus onset.

Event-related potentials were further interrogated with independent components analysis (fastICA; Hyvarinen, 1999) and minimum norm distributed source current density reconstruction (CDR). The pre-trigger interval (−500−0 ms) was used for noise estimation. Source localization was performed with a relative deviation of 100% and Lp Norm model, data = 2.0. A boundary element head model (BEM) based on the ICBM-152 T1 atlas was used with the following estimates for compartment thickness and conductivity: skin = 9 mm (0.3300 S/m), outer skull = 8 mm (0.0132 S/m), and inner skull (source surface) = 6 mm (0.3300 S/m) (Fuchs et al., 1998, 2001, 2002). The ICBM model contained 193 slices and was semi-automatically segmented with region growing and seedpoint identification based on intensity thresholds. The resulting projection space was comprised of 5667 nodes with 11322 triangles (Skin: 1765 nodes, 3526 triangles; outer skull: 1591 nodes, 3178 triangles; inner skull: 2311 nodes, 4618 triangles). Activation was displayed on a standard triangle mesh cortex (62080 triangles, side length = 2.5 mm, with normals) and localized in MNI SPM99 coordinate space (R/L, A/P, and S/I) with corresponding Brodmann Areas (BAs) reported where available.

Prior to CDR source localization with statistical low-resolution electromagnetic tomography (sLORETA; Pascual-Marqui, 2002), PCA was used to screen components with a low SNR (SNR ≤ 0.9); all components above this threshold were retained for ICA. For initial visualization, IC-based sources were calculated on GA WFs for each treatment and timepoint after down-sampling the data from 2000Hz to 250Hz. A 50% intensity cut-off was applied to emphasize dominant sources. Based on the global GA WF for upper- and lower-body CRTs, regularization factors of λ = 351 and λ = 221 were used for upper- and lower-body stimuli, respectively (Wischmann et al., 1992). These regularization factors were then applied to individual ERP averages for statistical comparisons of source activity.

Event-related oscillations were examined with Fast Fourier Transforms (FFT) on grand- and individual-ERP averages for qualitative and statistical comparisons, respectively. Epochs (−500−1000 ms post-stimulus) were windowed with a Hann Filter (10% width) and binned from 0.5−4.0Hz (delta), 4.0−8.0Hz (theta), 8.0−14.0Hz (alpha), 14.0−30.0Hz (beta), and 30.0−40.0Hz (gamma). For each frequency range, activity was averaged by treatment and timepoint and qualitatively analyzed with (spherical spline expansion) interpolated scalp topography maps. Difference maps were calculated to explore the influence of exercise on global spectral activity. For insight into the timing of phase-locked frequency modulation, wavelet analysis was performed on GA WFs for midline and lateral electrode averages (Fz, Cz, Pz, Oz, C5-C6, and T7-T8) selected based on the results of quantitative ERP and FFT analyses (Mexican Hat Wavelet, resolution = 1.00 Hz). To improve the visibility of higher frequency activity, a frequency-weighted instantaneous energy operator was used (Kaiser, 1993).

### Statistical Analyses

Median response times were analyzed separately for upper- and lower-body CRTs with 3 treatment × 2 timepoint repeated measures ANOVAs and Sidak corrections for multiple comparisons (Prism 8, GraphPad Software, San Diego, CA, United States). Quantitative differences in sensor, source, and oscillatory activity were assessed for each segment with permutation statistics (statistical non-parametric mapping). A 3 treatment × 2 timepoint repeated measures analysis matrix assessed main effects for treatments, timepoints, and interactions (Wagner et al., 2017). For the analysis of ERPs and phase-locked oscillatory activity, based on a 2000 Hz sampling rate, 40Hz low-pass filtering frequency, and significance level of *p* ≤ 0.05, 24,395 and 1,000 randomizations were performed with multiple comparison-corrected significance levels of *p* = 0.002 and *p* = 0.05, respectively. Source activity was similarly analyzed after normalization and log-transformation (Wagner et al., 2017). Based on a 250 Hz sampling rate, 40Hz low-pass filtering frequency, and significance level of *p* ≤ 0.05, 3,071 randomizations were performed with a multiple comparison-adjusted significance level of *p* = 0.016. The cerebellum was excluded as a potential activity source. When a main effect or interaction was evident, differences in sensor or source activity were compared across group averages for treatment(s) and timepoints to determine simple effects.

## Results

The CRTs displayed stereotypic ERPs, source activities, and evoked oscillations; sensitivity to intense exercise; and task-specificity. The most salient cortical responses included: (1) P1-N1, P3 (upper-body), and RP (lower-body) ERP waveforms; (2) cortical source activity concentrated in the superior and medial frontal gyrus, superior and middle temporal gyrus, and task-specific regions of sensorimotor cortex; and (3) evoked delta and theta oscillatory activity at frontocentral, occipital, and temporal leads, alpha activity at parieto-occipital leads, and beta/gamma activity concentrated at frontotemporal and occipital leads.

### CRT Response Times

Response times improved after exercise for upper- (−12.25 ± 3.48 ms from pre- to post-exercise, *F*(1,18) = 12.42, *p* = 0.002 (95%CI[4.95−19.6 ms])) and lower-body CRTs (−12.29 ± 4.85 ms, *F*(1,18) = 6.41, *p* = 0.021, (95%CI[2.09−22.48])) (Table 2). Response times did not differ by treatment [upper: *F*(1.7,31.4) = 1.04, *p* = 0.357; lower: *F*(1.9,33.5) = 0.58, *p* = 0.553] and there was no interaction between treatment and timepoint (upper: *F*(1.6,29.5) = 1.04, *p* = 0.354; lower: *F*(1.7,30.2) = 0.05, *p* = 0.926).

**TABLE 2:** Median response times by segment, treatment, and timepoint.

		**Response Time (ms)**		
**Segment**	**Timepoint**	**PLA**	**160**	**960**
Upper	Pre	435.4 ± 34.8	440.7 ± 48.3	448.3 ± 47.1
	Post	423.7 ± 38.2 *	433.7 ± 33.9 *	430.3 ± 35.9 *
Lower	Pre	776.3 ± 60.9	787.0 ± 74.6	778.1 ± 45.8
	Post	763.7 ± 70.6 *	773.0 ± 66.9 *	767.9 ± 52.2 *

Values are grand means ± SD; *Improved response time compared to pre-exercise (*p* ≤ 0.05).

### Event-Related Potentials

Upper- and lower-body CRTs produced stereotypic ERP waveforms and topographies (Figure 2). Amplitudes and latencies of hypothesized components for each segment, treatment, and timepoint GA WF are provided in Table 3. For the upper-body, parietal (negative) and frontotemporal (positive) sensor polarity increased around 150−170 ms, followed by deflections in frontocentral and parietal leads from 240−315 ms post-stimulus. Around response execution, a bilateral increase in central positivity was evident. For the lower-body CRT, increased polarity was similarly evident around 160 and 250 ms post-stimulus in parietal/frontotemporal and frontocentral/parietal leads, respectively, but preceded by increased central positivity surrounding stimulus presentation. In addition, as soon as 250 ms post-stimulus, increased central negative polarity dominated until response execution. As the onset and offset of central positivity around stimulus response (upper-body) and presentation (lower-body) times could not be reliably defined, these waveforms were excluded from further analysis.

**TABLE 3:** Amplitude and latency for ERPs by segment, treatment, and timepoint.

			**PLA**		**160**		**960**	
**Segment**	**ERP (Sensor)**	**Measure**	**Pre**	**Post**	**Pre**	**Post**	**Pre**	**Post**
Upper	P1 (F8)	Amplitude (uV⋅ms)	820.2	565.5	686.3	553.1	628.7	325.7
		Latency (ms)	139.5	134.0	143.0	138.0	146.5	144.0
	N1 (P2)	Amplitude	−602.6	–508.9	–617.4	–552.0	–773.1	–381.3
		Latency	142.0	138.0	141.0	141.5	141.5	144.0
	P3a (Pz)	Amplitude	572.6	409.3	571.7	500.1	721.7	443.4
		Latency	231.5	228.5	232.0	232.5	233.5	232.0
	P3b (CPz)	Amplitude	1461.1	1026.5	1267.5	1019.0	1604.7	986.0
		Latency	287.5	273.0	282.5	283.0	278.0	285.5
Lower	P1 (F8)	Amplitude	832.2	544.1	728.1	603.9	751.9	368.5
		Latency	141.5	139.5	142.0	144.5	142.5	137.5
	N1 (P2)	Amplitude	–576.5	–572.7	–813.0	–641.3	–796.2	–562.3
		Latency	143.0	143.5	145.0	144.5	145.5	142.5
	P2/P3a (PO4)	Amplitude	708.0	568.5	651.6	536.1	800.3	535.1
		Latency	210.5	210.5	212.0	210.0	215.5	211.0
	RP (Cz)	Amplitude	–5761.2	–5291.9	–6088.5	–4951.7	–6693.6	–5629.1
		Latency	369.0	377.5	376.5	360.0	384.5	377.0

For quantitative comparisons of ERP topographies, homogeneity of variance was indicated by significant consistency over the analysis time range (Wagner et al., 2017). There were no main effects for treatment overall. Examination of grand-averaged ERP latencies revealed a reduction in P1-N1 latency after exercise with 960mg (Figure 2B), and a dose-dependent increase in P3b latency for the upper body (Figure 2C). More generally, ERP amplitudes were lower after exercise (Figure 2D).

### Independent Component-Based Current Density Reconstructions

**FIGURE 3:**
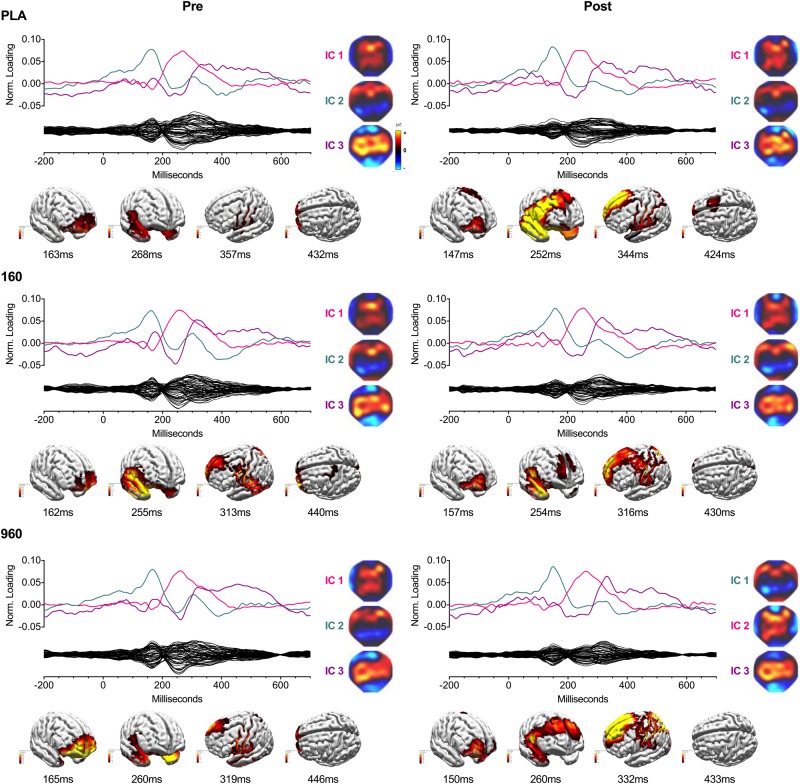
IC Waveforms and CDRs for Upper-Body Stimuli. Three ICs were identified from grand-averaged 64-channel ERP waveforms. For each treatment and timepoint, IC waveforms are depicted in the upper axis and matched by color and ERP correspondence, with the IC-filtered grand average waveform depicted on the lower axis. Spherical spline interpolated 2D scalp load distribution maps for each component are depicted to the right of the waveforms. IC-based sLORETA CDR localizations are provided at the bottom of each panel (common average reference). The displayed perspectives emphasize areas of maximal activation common to each treatment and timepoint. Intensities of 2D maps and source plots are scaled in relation to pre-exercise placebo values for each component (maps) or timepoint (sources). For source plots, activation was clipped at 50% to emphasize the most prominent sources. Source activity is displayed in pseudo-F scale units, with a regularization factor of λ = 351. ERP, event-related potential; IC, independent component; sLORETA, standardized low-resolution brain electromagnetic tomography; CDR, current density reconstruction.

Independent components analysis of upper-body CRT GA WFs revealed three consistent ICs, which corresponded to a P1-N1-like waveform (typically IC2) with a 150−165 ms peak above frontotemporal (positive) and parieto-occipital (negative) sites, P3a (typically IC1) that peaked around 250−280 ms above frontocentral sites, and P3b that peaked diffusely above central sites around 300−330 ms (IC3) (Figure 3). Independent component-based CDRs for each treatment and timepoint were examined at the corresponding global field power (GFP) peaks and median response times and indicated: (1) superior/medial frontal gyrus (BA 10) origin for IC2; (2) superior temporal gyrus and superior/middle/inferior frontal gyrus (BA 8/9) sources for IC1; (3) superior/middle frontal gyrus (BA 8/9), sensorimotor (BA 1-4), insula (BA 13), superior/middle/parahippocampal temporal gyrus (BA 21/22/35) and precuneus (BA 7) sources for IC3; and (4) superior/medial frontal (BA 10) and precentral (BA 6) gyrus sources at the median response time.

**FIGURE 4:**
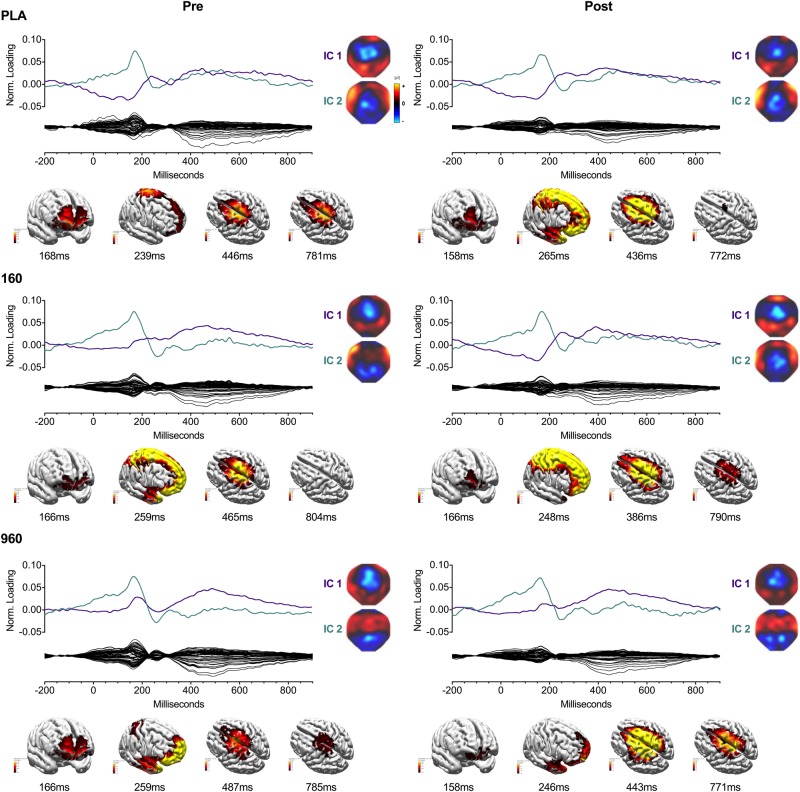
IC Waveforms and CDRs for Lower-Body Stimuli. Two ICs were identified from the grand-averaged 64-channel ERP waveforms. For each treatment and timepoint, IC waveforms are depicted in the upper axis and matched by color and ERP correspondence, with the IC-filtered grand average waveform depicted on the lower axis. Spherical spline interpolated 2D scalp load distribution maps for each component are depicted to the right of the waveforms. IC-based sLORETA CDR localizations are provided at the bottom of each panel (common average reference). The displayed perspectives emphasize areas of maximal activation common to each treatment and timepoint. Intensities of 2D maps and source plots are scaled in relation to pre-exercise Placebo values for each component (maps) or timepoint (sources). For source plots, activation was clipped at 50% to emphasize the most prominent sources. Source activity is displayed in pseudo-F scale units, with a regularization factor of λ = 221. ERP, event-related potential; IC, independent component; sLORETA: standardized low-resolution brain electromagnetic tomography; CDR, current density reconstruction.

Lower-body CRT GA WFs were associated with two consistent ICs (Figure 4). The earliest component (IC2) peaked around 150 ms with a positive frontotemporal and negative centroparietal scalp distribution. Similar to the upper body, source localization delimited IC2 activity to the superior/medial frontal gyrus (BA 10). The later component (IC1) was a readiness potential, characterized by frontocentral negativity from ∼230 ms to the point of response initiation. Activity sources were concentrated in the sensorimotor cortex (M1/S1) and superior frontal gyrus (BA 6). For both CRTs, cross-comparison of peak activity times confirmed that differences in activation did not reflect variation in IC peak latency. Information on percentage of variance explained, SNRs, and temporal properties of ICs is provided in Table 4.

**TABLE 4:** Independent component analysis by treatment, segment, and timepoint.

			**Component**								
			**1**			**2**			**3**		
**Treatment**	**Segment**	**Timepoint**	**% Var**	**SNR**	**Peak (ms) (SNR)**	**% Var**	**SNR**	**Peak (ms) (SNR)**	**% Var**	**SNR**	**Peak (ms) (SNR)**
PLA	Upper	Pre	25.8	1.4	166 (–0.72), 268 (4.30)	21.0	1.1	161 (3.65), 414 (–1.22)	12.5	0.7	225 (–0.78), 357 (1.26)
		Post	23.7	1.5	233 (4.90), 446 (–0.77)	18.5	1.2	147 (4.24), 404 (–1.24)	13.1	0.8	225 (–1.14), 321 (1.81)
	Lower	Pre	23.7	1.5	140 (–2.52), 446 (2.57)	19.7	1.3	170 (4.50), 267 (–0.48)			
		Post	24.0	1.5	141 (–2.30), 431 (2.58)	18.2	1.1	161 (3.53), 256 (–0.75)			
160	Upper	Pre	27.7	1.6	166 (–1.10), 255 (5.03)	20.1	1.1	161 (3.58), 387 (–1.77)	11.8	0.7	243 (–1.33), 313 (1.50)
		Post	20.3	1.1	252 (3.55), 443 (–0.63)	19.2	1.0	156 (3.34), 399 (–1.38)	13.8	0.7	217 (–0.87), 316 (1.77)
	Lower	Pre	23.3	1.4	–17 (–0.57), 463 (2.95)	22.4	1.4	167 (4.84), 261 (–1.63)			
		Post	24.7	1.5	147 (–2.50), 388 (2.98)	19.5	1.2	169 (3.41), 262 (–0.73)			
960	Upper	Pre	26.3	1.6	163 (–0.68), 260 (5.09)	23.4	1.4	165 (4.70), 389 (–1.51)	10.7	0.6	261 (–0.91), 450 (1.26)
		Post	21.1	1.3	150 (4.62), 372 (–1.12)	20.3	1.2	120 (–0.62), 260 (3.90)	11.4	0.7	198 (–0.79), 332 (1.87)
	Lower	Pre	24.5	1.6	68 (–0.42), 488 (3.51)	24.0	1.5	166 (5.38), 259 (–2.01)			
		Post	25.2	1.5	–8 (–0.67), 442 (3.29)	18.1	1.1	161 (3.66), 245 (–1.12)			

*SNR, signal-to-noise ratio.*

### Source Activity Analysis

For the upper-body, there were treatment-specific differences in current density from 220−264 ms in the postcentral gyrus (BA 43: 68.7, −7.7, 15.6), superior temporal gyrus (BA 22: 69.5, −8.8, −1.7), and middle temporal gyrus (BA 21: 68.9, −10.8, −19.7) (*F* = 14.6, *p* = 0.000). Further analysis resolved these differences to placebo, which had less activity compared to 160 and 960 mg. Analysis of treatment by timepoint interactions revealed no effects beyond those expected due to randomization.

For lower-body CRTs, an interaction between treatment and timepoint was evident, with greater post-exercise activity from −192 to −180 ms in the medial frontal gyrus (−4.1, 45.6, 28.1), superior frontal gyrus (BA 8: −4.1, 53.9, 35.8), and anterior cingulate (BA 32/24: −4.1, 36.5, 10.6) after 160 and 960 mg treatments compared to Placebo (*F* = 5.4, *p* = 0.001). From 76−84 ms, post-exercise activity in the superior frontal gyrus (40.5, 28.5, and 47.7) increased for 960 mg compared to 160 mg and placebo (*F* = 4.3, *p* = 0.006). Visualized main effects and interactions for upper and lower body CRTs are provided in Supplementary Material (Supplementary Figure S1).

### Event-Related Oscillations

Visual examination of grand-averaged phase-locked oscillatory activity revealed spatially heterogeneous patterns based on frequency (see Supplementary Figure S2). Delta and theta activity were concentrated at frontocentral, occipital, and temporal leads, with alpha at parieto-occipital leads, and beta/gamma at frontotemporal and occipital leads. Statistical analysis of FFT spectral activity revealed a generalized reduction in event-related oscillatory activity after exercise, but some features varied depending on segment and treatment. Given significant interactions between treatments and timepoints, main effects for treatment are not presented. Time-varying phase-locked spectral activity was visualized using spectrograms (Supplementary Figures S3–S8).

**FIGURE 5:**
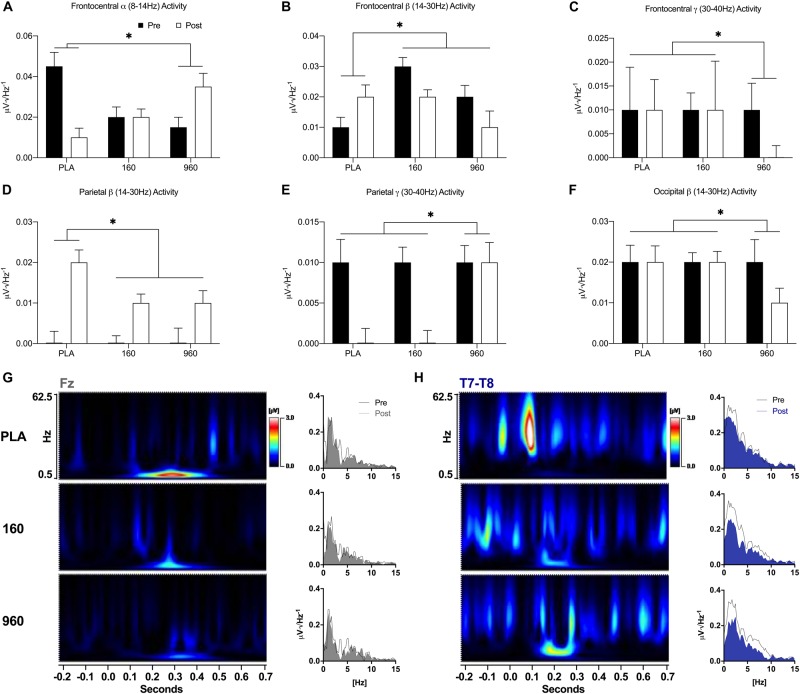
Event-Related Oscillatory Activity for Upper-Body Stimuli. **(A–F)** FFTs were applied to ERPs from –500 to 1000 ms with a Hann Filter (10% width). Spectral activity was binned from 0.5 to 4.0 Hz (delta), 4.0 to 8.0 Hz (theta), 8.0 to 14.0 Hz (alpha), 14.0 to 30.0 Hz (beta), and 30.0 to 40 Hz (gamma). Each plot depicts grand-averaged spectral activity (mean ± SE) for regions with statistical interactions between treatment and timepoint (^∗^ = *p* ≤ 0.05). Black and open bars indicate pre- and post-exercise values, respectively. **(G–H)** Time-varying and overall phase-locked spectral activity was examined with spectrograms and spectrum plots of grand-averaged ERPs using wavelet analysis (Mexican Hat, resolution = 1.0Hz, Teager-Kaiser, 0.5–62.5 Hz activity, and –200 to 700 ms) and FFTs for midline (Fz, Cz, Pz, and Oz) and lateral electrode averages (C5–C6 and T7–T8) based on the results of quantitative ERP and FFT analyses. Spectrogram activity is interpolated and scaled against pre-exercise placebo values specific to each sensor. For sensors that displayed qualitative dose-dependent responses (Fz and T7–T8), spectrograms are difference plots (pre- minus post-exercise activity) while the spectrum plots display 0–15 Hz activity with pre- and post-exercise activity overlaid. FFT, fast Fourier transform; ERP, event-related potential.

During the upper-body CRT, frontocentral alpha activity was lower after exercise for placebo and similar or increased for 160 and 960 mg, respectively (*F* = 4.1, *p* = 0.027, Figure 5A). Frontocentral beta activity increased after exercise for placebo, but decreased for active treatments (*F* = 6.7, *p* = 0.006, Figure 5B). Frontocentral gamma activity was similar for placebo and 160mg after exercise, but lower for 960mg (*F* = 5.5, *p* = 0.037, Figure 5C). There was a generalized increase in parietal beta activity after exercise, with smaller responses for active treatments (*F* = 6.5, *p* = 0.021, Figure 5D). At right parietal leads, post-exercise gamma activity was lower for placebo and 160 mg but similar for 960 mg (*F* = 6.5, *p* = 0.020, Figure 5E). Occipital beta activity was reduced after exercise with 960 mg, but similar for placebo and 160mg (*F* = 7.1, *p* = 0.006, Figure 5F). Time-frequency analysis revealed a dose-dependent reduction in frontal-midline theta (FM-theta, Fz) activity at 275 ms, with 960mg retaining the most activity (Figure 5G). There was also a dose-dependent reduction in temporal theta activity from 150−250 ms (largest for 960 mg, Figure 5H).

**FIGURE 6:**
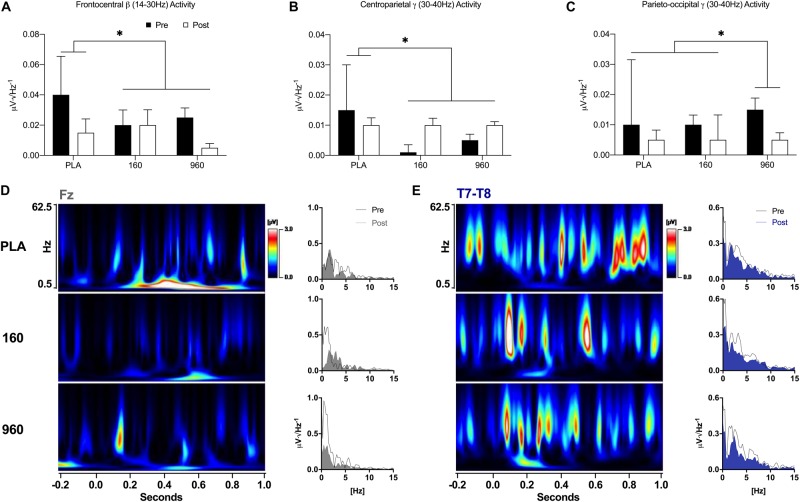
Event-Related Oscillatory Activity for Lower-Body Stimuli. **(A–C)** FFTs were applied to ERPs from –500 to 1000 ms with a Hann Filter (10% width). Spectral activity was binned from 0.5–4.0 Hz (delta), 4.0–8.0 Hz (theta), 8.0–14.0 Hz (alpha), 14.0–30.0 Hz (beta), and 30.0–40 Hz (gamma). Each plot depicts grand-averaged spectral activity (mean ± SE) for regions with statistical interactions between treatment and timepoint (^∗^ = *p* ≤ 0.05). Black and open bars indicate pre- and post-exercise values, respectively. **(D–E)** Time-varying and overall phase-locked spectral activity was examined with spectrograms and spectrum plots of grand-averaged ERPs using wavelet analysis (Mexican Hat, resolution = 1.0Hz, Teager-Kaiser, 0.5–62.5Hz activity, –200 to 1000 ms) and FFTs for midline (Fz, Cz, Pz, and Oz) and lateral electrode averages (C5-C6 and T7-T8) based on the results of quantitative ERP and FFT analyses. Spectrogram activity is interpolated and scaled against pre-exercise placebo values specific to each sensor. For sensors that displayed qualitative dose-dependent responses (Fz and T7-T8), spectrograms are difference plots (pre- minus post-exercise activity) while the spectrum plots display 0–15 Hz activity with pre- and post-exercise activity overlaid. FFT, fast Fourier transform; ERP, event-related potential.

During lower-body CRTs, placebo displayed greater reductions in frontocentral beta activity after exercise compared to active treatments (*F* = 5.9, *p* = 0.021, Figure 6A). At centroparietal sites, gamma activity was reduced after exercise with placebo but increased with active treatments (*F* = 5.7, *p* = 0.024, Figure 6B). At parieto-occipital locations, gamma activity was more strongly reduced after exercise with 960mg (*F* = 5.7, *p* = 0.027, Figure 6C). In the right temporal area, placebo displayed the largest post-exercise reduction in beta activity (*F* = 5.1, *p* = 0.038). Time-frequency wavelet analysis indicated that greater frontal delta activity for active treatments before exercise was accompanied by larger reductions afterward with similar or increased activity for placebo (Figure 6D). Conversely, placebo displayed greater reductions in FM-theta activity after exercise. Similar to the upper body, there was a dose-dependent reduction in temporal theta activity after exercise (Figure 6E).

## Discussion

The dose-dependent effect of CK on psychomotor cognitive function was assessed based on performance and cortical activity during upper- and lower-body CRTs completed before and after intense lower-body exercise. Ginsenoside supplementation did not improve response speed at rest or augment generalized improvements after exercise. Yet differences in evoked potential latencies, spectral properties, and IC-based source activities indicated improvements in attentional functions and moderation of exercise-induced arousal. Responses to upper- and lower-body CRTs also indicated task-specific effects on psychomotor cognition.

A higher dose (960 mg) of CK reduced P1/N1 latencies during lower-body CRTs, which might improve visual stimulus discrimination by counteracting reductions in sensitivity to exogenous sensory inputs after exercise, given generalized reductions in P1−N1 amplitude and conceptions of the P1−N1 as an index of perceptual aspects of selective attention (Luck and Kappenman, 2013). During upper-body CRTs, dose-dependent increases in P3b latency after exercise could indicate that a higher dose of CK impaired processing times related to the allocation of attentional resources (Polich, 2007; Kumar et al., 2010). Nevertheless, given the sensitivity of P3b to autonomic inputs (Nieuwenhuis et al., 2005) and CK-induced reductions in circulating cortical after exercise (Flanagan et al., 2018), relative increases in P3b latency may reflect reductions in exercise-induced sympathetic activity (Kubota et al., 2001).

Source activity during the P3 interval was concentrated in frontal and temporoparietal brain regions with additional occipital and sensorimotor activity indicative of visuomotor task demands (Polich and Comerchero, 2003). Treatment-specific differences in source activity were limited to the upper-body CRT, with CK supplementation accompanied by greater activity in the postcentral gyrus and medial/superior temporal gyrus. After exercise, there was less activity around response initiation in areas of the postcentral gyrus corresponding to the upper extremities. Given reduced activity in the anterior cingulate at the same timepoint, diminished activity in S1 and other primary regions of cortex could reflect the diversion of attention from task-relevant stimuli to sensory processes associated with fatiguing lower-body exercise.

The results of spectral analysis complemented those of IC-based ERPs. The direct relationship between treatment dosage and frontal alpha activity after exercise supports the conclusion that CK improved top-down attentional control processes after fatiguing lower-body exercise (Klimesch et al., 2007; Klimesch, 2012). Dose-dependent reductions in FM-theta activity (960mg retained the most activity) also suggest that CK improved electrophysiological markers of sustained attention or working memory functions (Holroyd and Umemoto, 2016). Opposing changes in temporal theta activity are notable because of the indirect relationship between hippocampal and FM-theta activity in non-human models (Mitchell et al., 2008). While different from the time course of FM-theta activity, dose-dependent increases in medial/superior frontal gyrus and anterior cingulate activity after exercise may explain the improvement of attentional processes (Ishii et al., 1999).

Regardless of the musculature used to perform CRTs, there was a reduction in frontal delta activity after exercise, yet for the lower-body during the majority of the post-stimulus interval activity appeared similar after placebo. Delta oscillations are directly related to arousal (Guntekin and Basar, 2016), which suggests that CK attenuated task-specific arousal after exercise. As a point of speculation, the alteration of delta activity by CK is notable because ginsenosides act on cholinergic neurotransmission (Lee et al., 2010; Wang et al., 2010), which mediates delta oscillations (Steriade, 1993; Steriade et al., 1993). Alternatively, the maintenance of frontal delta activity with placebo may explain equal behavioral performance if such activity was reflective of internal efforts to reduce the influence of sensory inputs that could interfere with concentration or task completion (Harmony, 2013).

Besides the effects CK on event-related activity related to arousal and attentional processes, a number of additional cortical responses were task-specific. During the upper-body CRT, CK was accompanied by comparative reductions in frontocentral and occipital beta activity, which supports the conclusion that CK mitigated arousal, cognitive load, or multisensory stimulation from task-irrelevant areas of cortex after exercise (Guntekin and Basar, 2014). At gamma frequencies, reduced frontocentral activity and preserved parietal activity may reflect improvements in local sensory integration with a higher CK dosage (Jensen et al., 2007). Conversely, during lower-body CRTs, placebo displayed the largest post-exercise reduction in frontocentral and right temporal beta activity, whereas active treatment was accompanied by similar or increased gamma activity at centroparietal sites. Collectively, these divergent responses may reflect attentional processes related to task-specific sensorimotor inputs.

While CK did not substantially affect movement-related cortical activity, segment-specific differences in readiness potentials (RPs: indicative of movement selection and preparation; Hallett, 1994; Toma et al., 2002) were evident and may explain divergent brain activity patterns during intermediate and late response stages. For example, the prominence of readiness potentials (RPs) during the lower-body CRT likely explain the attenuation (P3a) or absence (P3b) of P3 components that occur at similar time and sensor space. The absence of RPs during upper-body CRTs may reflect subtle differences in task execution, such as the production of tonic hand activity during the upper-body CRT versus relative inactivity of the legs between responses during the lower-body CRT. Nevertheless, it is notable that lower-body RPs were delimited principally to dorsal regions of sensorimotor cortex where the leg representations are contained. Alternatively, in contrast to upper-body CRTs where each stimulus and response occurred at the same location, lower-body responses were made at a location that was not visually attended, as participants were instructed to avoid looking at the underlying press pad to minimize movement-related artifacts. If additional visuospatial resources were required to compensate for exercise-induced fatigue in the lower-body, this subtle difference between tasks might explain increased activity in the precuneus and posterior cingulate around response times during lower-body CRTs.

Given global reductions in ERP and phase-locked spectral activity, intense exercise appeared to induce a transient increase in cortical activation. This contention is supported by recent work that used interleaved TMS-EEG to demonstrate increased corticospinal excitability and decreased GABA_*B*_-dependent inhibition after fatiguing exercise (Otieno et al., 2019). The effects of fatiguing exercise were frequently lateralized in the right hemisphere, where the frontal-parietal attention network is most prominent (Posner and Dehaene, 1994). In addition, reductions in occipital activity specific to the lower-body corresponded with decreases in delta activity. Given the relationship between delta activity, arousal, and task-related brain activity, lower-body exercise appeared to reduce visual processing capacity, but only during a lower-body visuomotor task. The influence of exercise on cortical source activity was largely restricted to thalamic, basal ganglia, and deeper limbic structures. If verified in future work with complementary neuroimaging techniques, these observations could provide important clues about the influence of exercise on brain function. Nevertheless, given the limitations of EEG source localization, activity in small and deep structures should be interpreted with extreme caution.

In the present study, cognitive responses to CK and exercise were assessed using a number of EEG analysis techniques. The relative contribution of evoked neural responses versus phase resetting to ERPs and oscillatory responses is beyond the scope of this investigation. Nevertheless, examination of induced and evoked activity on a single trial basis may provide additional insights into the nature of CK’s effects on cognition. Given the combination of a large number of sensors and endpoints the use of permutation statistics, conservative adjustments for multiple comparisons, a smaller number of statistical comparisons, and analysis of the entire signal interval and electrode montage minimized false positives and the risk of bias (Luck and Gaspelin, 2017). Nevertheless, there are important limitations to consider. As head surface coverage did not extend inferior to the axial plane, source estimation involving ventral and deeper aspects of the brain must be interpreted cautiously. In addition, techniques such as electrode digitization and individualized head models may improve the accuracy of source localization in future work. Thirdly, responses to the CRT may generalize to other cognitive domains but task- and population-specific responses are likely and require clarification before any overarching conclusions about the influence of CK on cognition are made.

In addition to dose-dependent reductions in perceived exertion and circulating cortisol after intense lower-body exercise (Flanagan et al., 2018), CK increased activity in cortical regions responsible for sustained attention while mitigating exercise-induced increases in arousal. Responses to exercise varied depending on task, but CK appeared to reduce sensory interference from lower-body exercise during an upper-body choice reaction test and improve the ability to maintain task-specific sensory processes. Our findings indicate that the effects of CK on brain activity are primarily inducible, and that cognitive responses may also involve indirect peripheral actions, such as the reduction of stress-induced sympathetic activity.

## Data Availability Statement

The raw data supporting the conclusions of this article will be made available by the authors, without undue reservation, to any qualified researcher.

## Ethics Statement

The studies involving human participants were reviewed and approved by The Ohio State University Office of Compliance and Institutional Review Board for use of human subjects in research. The patients/participants provided their written informed consent to participate in this study.

## Author Contributions

SF and WK contributed to conception and design of the study. SF and FP performed the data and statistical analysis. SF wrote the first draft of the manuscript. CD-L, MC, AS, and CC contributed to data interpretation. All authors contributed to the manuscript revision, and read and approved the submitted version.

## Conflict of Interest

The authors declare that the research was conducted in the absence of any commercial or financial relationships that could be construed as a potential conflict of interest.
