# Supplementary material for: Constitutive and Stress-Induced Psychomotor Cortical Responses to Compound K Supplementation
Source: Front Neurosci. 2020 Apr 8;14:315. doi: 10.3389/fnins.2020.00315 (PMC7158875; doi:10.3389/fnins.2020.00315)
Supplement: Supplementary file 1 [file Image_1.pdf]

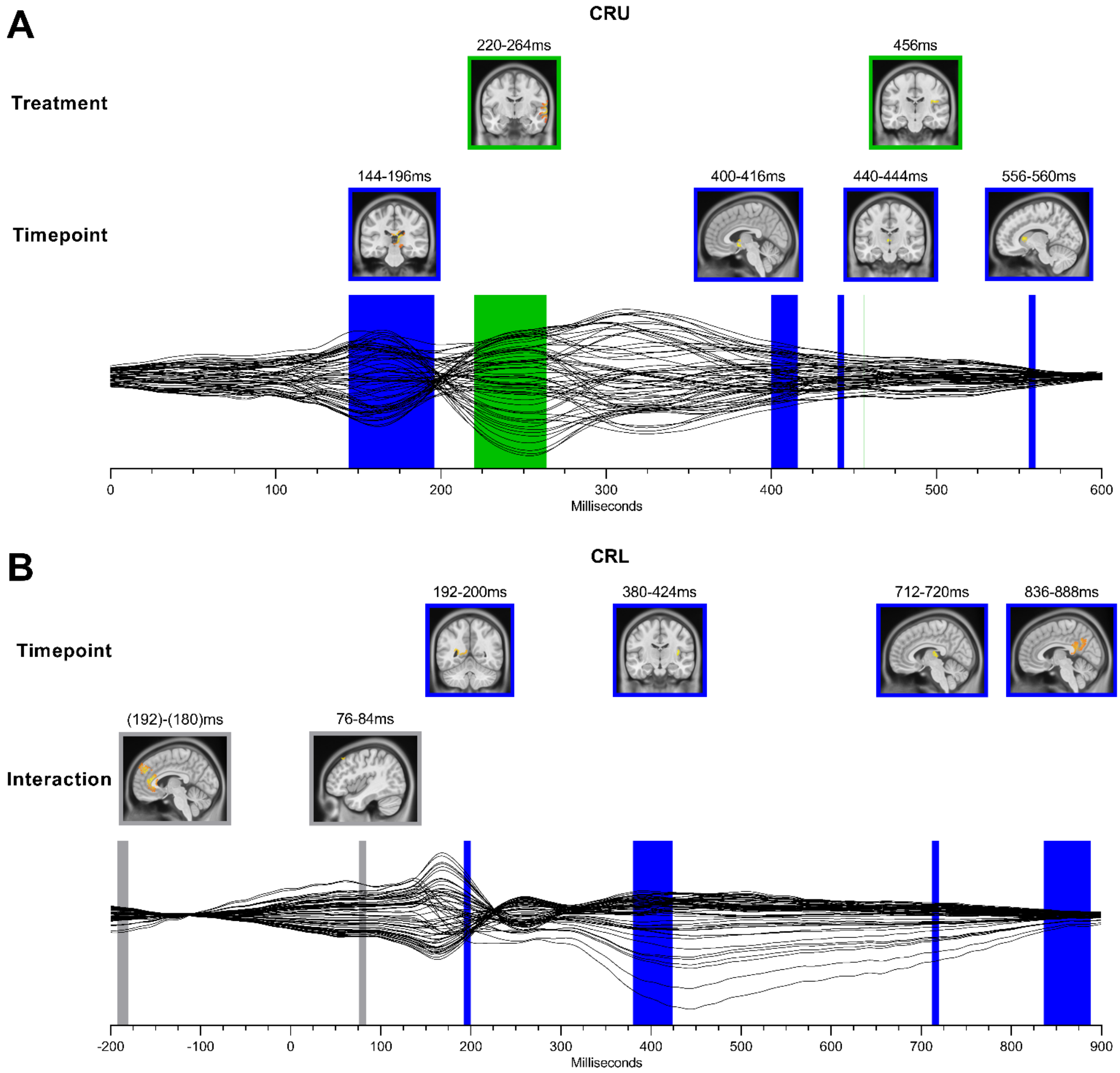

Supplementary Figure 1. sLORETA Statistics for Upper- and Lower-Body Stimuli. Non-parametric permutation tests indicated differences in A) upper- and B) lower-body CRT source activity for treatments (green), timepoints (blue), and treatment x timepoint interactions (grey). Timing of differences is indicated above each image and highlighted areas of the grand-averaged IC-filtered ERP waveform depicted at the bottom of each panel. For each segment, a 250Hz sampling rate, 40Hz low pass filter, and initial significance level of $p \leq 0.05$ resulted in 3,071 randomizations with a multiple-comparison-adjusted significance level of $p=0.016$. For coronal images, the right hemisphere is on the right. For the upper body, from 144-196ms, there was decreased activity in the medial dorsal nucleus (6.2, -22.0, 6.0) and posterior cingulate (BA 23: 6.2, -36.5, 25.0) after exercise ($F=22.2$, $p=0.001$). From 400-416ms, activity was lower in the anterior cingulate (BA 25: 4.8, 5.9, -11.0) and postcentral gyrus (18.9, -42.1, 73.7) ($F=17.7$, $p=0.006$). From 440-444ms, activity increased in the medial dorsal nucleus (-2.2, -16.7, 1.7) ($F=17.3$, $p=0.016$). From 556-560ms, there was a reduction in activity in the caudate body (11.9, 15.7, 3.8) ($F=19.0$, $p=0.007$). For the lower body, there were also a number of generalized responses to exercise, including: 1) less activity in the parahippocampal gyrus from 192-200ms (BA 30: -10.0, -47.5, 2.9) ($F=17.6$, $p=0.004$); 2) less activity in the insula from 380-424ms (38.4, -14.6, 7.1) ($F=16.6$, $p=0.004$); 3) increased activity in the medial dorsal nucleus from 712-720ms (4.1, -16.0, 3.6) ($F=15.9$, $p=0.010$); and 4) increased activity in the precuneus (BA 31: 5.5, -62.2, 30.2) and posterior cingulate from 836-888ms (BA 30: 5.5, -46.1, 22.5) ($F=21.6$, $p=0.002$). sLORETA: statistical low-resolution electromagnetic tomography; CRT: choice reaction test; ERP: event-related potential.
