# Supplementary material for: Constitutive and Stress-Induced Psychomotor Cortical Responses to Compound K Supplementation
Source: Front Neurosci. 2020 Apr 8;14:315. doi: 10.3389/fnins.2020.00315 (PMC7158875; doi:10.3389/fnins.2020.00315)
Supplement: Supplementary file 2 [file Image_2.pdf]

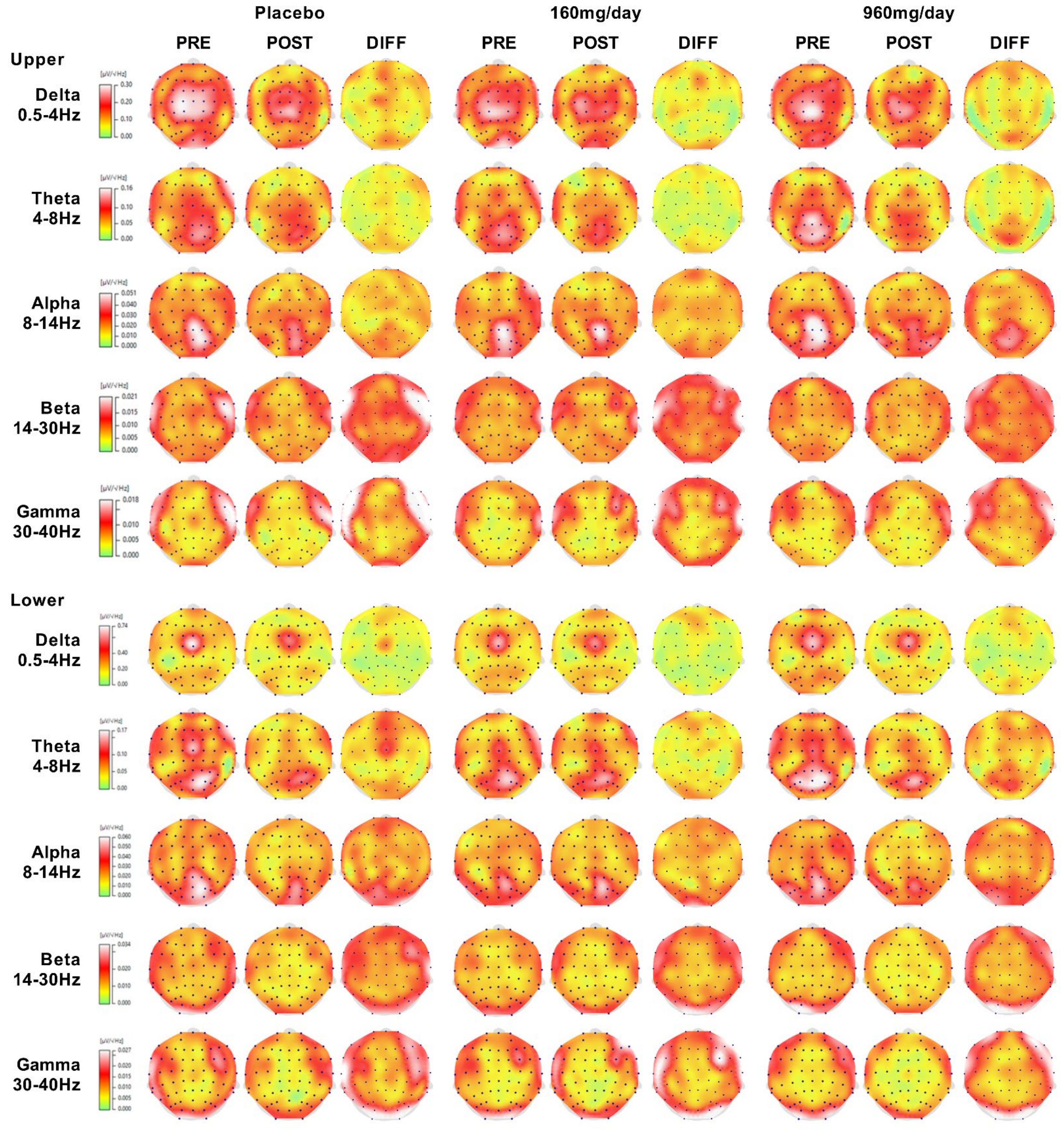

Supplementary Figure 2. Spectral Scalp Topographies for Upper- and Lower-Body Grand Average Waveforms. Fast Fourier Transforms (FFT) were applied to grand-averaged ERP waveforms from -500 to 1000ms with a Hann Filter (10% width). Spectral activity was binned from 0.5-4.0Hz (delta), 4.0-8.0Hz (theta), 8.0-14.0Hz (alpha), 14.0-30.0Hz (beta), and 30.0-40Hz (gamma). Average activity for each bin is displayed for each segment, treatment, and timepoint using 2D scalp topography maps with spherical spline interpolation. For each condition and frequency, differences between pre- and post-exercise activity are displayed using difference plots. For each frequency range, activity is scaled in relation to Placebo at the pre-exercise timepoint. ERP: event-related potential.

For upper-body CRTs, in the delta range, activity reductions were most pronounced at frontal (F=14.4-39.9, p=0.033-0.001) and right temporal leads (F=23.5-25.4, p=0.019). In the theta band, reductions were more diffuse, and included frontal (F=16.8-36.1, p=0.015-0.001), central (F=13.8-20.0, p=0.009-0.004), right temporal (F=18.6-25.8, p=0.004-0.001), and parieto-occipital (F=18.6-23.0, p=0.001) leads. Reductions in alpha activity were concentrated at right frontal (F=17.4-21.7, p=0.024-0.010) and occipital (F=20.2-27.4, p=0.001) electrodes. Beta activity reductions were less focal but concentrated at frontocentral (F=22.9-24.8, p=0.037-0.021), temporal (F=22.2, p=0.039), and parieto-occipital (F=21.2-21.9, p=0.048-0.021) leads. Gamma activity reductions were concentrated at frontocentral (F=23.0-27.9, p=0.038-0.010) and temporal (F=24.0-28.3, p=0.025-0.022) sites.

For lower-body CRTs, delta activity reductions were concentrated at frontocentral (F=16.2, p=0.041) and bilateral temporal sites (F=17.8-34.3, p=0.003-0.001). Theta frequency was similarly reduced at frontocentral (F=28.4, p=0.004) and bilateral temporal (F=18.0-19.6, p=0.004-0.002) leads, with additional reductions at parieto-occipital sites (F=19.9-22.5, p=0.004). Alpha activity reductions were more diffuse and included frontal (F=16.7, p=0.038), central (F=23.3-31.0, p=0.002), bilateral temporal (F=19.0-28.1, p=0.029-0.002) and occipital (F=18.5, p=0.029) electrodes. Beta activity was reduced at frontal sites (F=20.7, p=0.039). Gamma activity reductions were concentrated bilaterally at temporal sites (F=21.8-27.0, p=0.005-0.020).
