# Supplementary material for: Constitutive and Stress-Induced Psychomotor Cortical Responses to Compound K Supplementation
Source: Front Neurosci. 2020 Apr 8;14:315. doi: 10.3389/fnins.2020.00315 (PMC7158875; doi:10.3389/fnins.2020.00315)
Supplement: Supplementary file 3 [file Image_3.pdf]

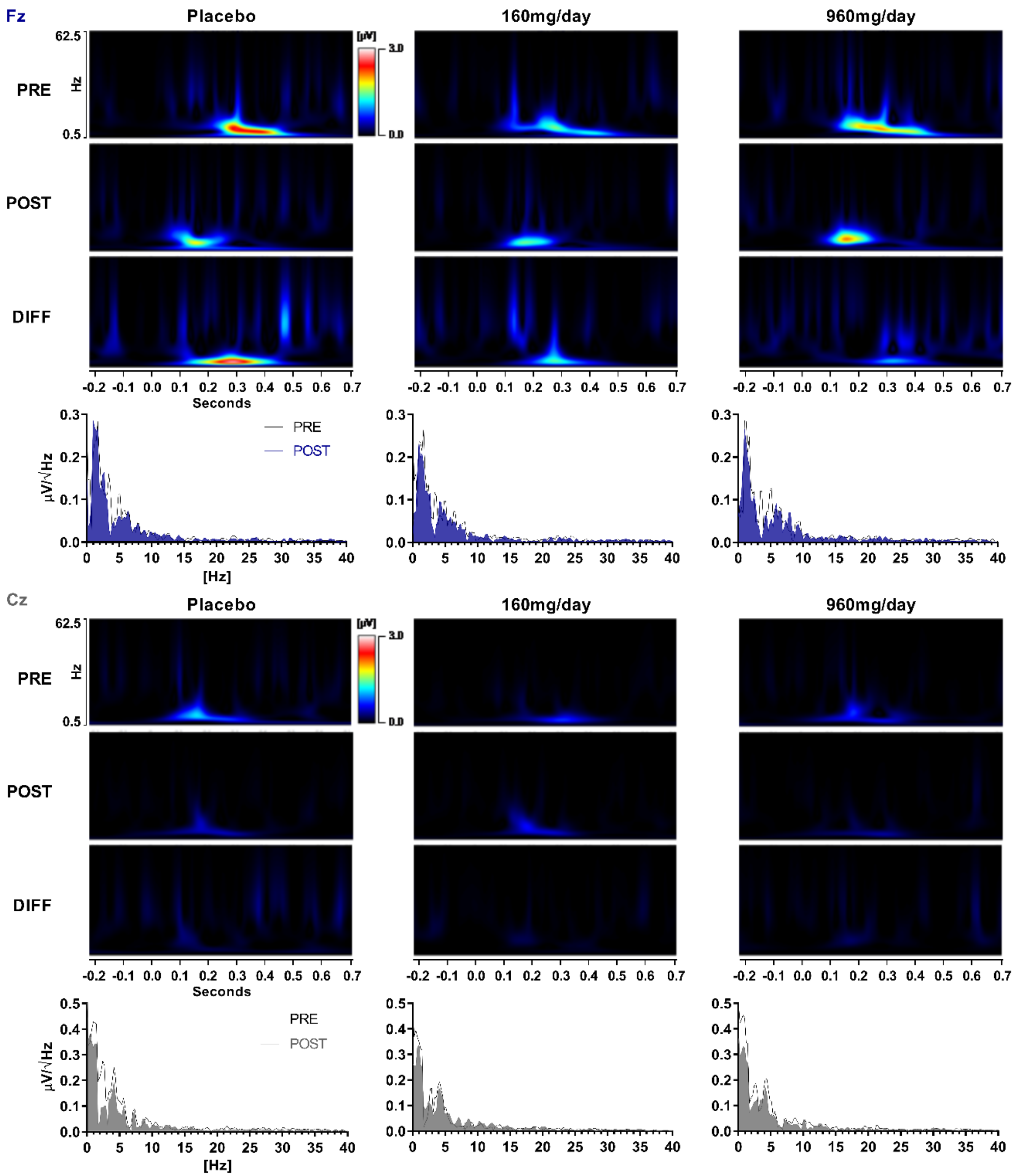

Supplementary Figure 3. Grand-Averaged Time-Frequency Spectrograms for Upper-Body Stimuli. Phase-locked spectral activity was examined with spectrograms of grand-averaged ERP waveforms using wavelet analysis (Mexican Hat, resolution = 1.0Hz, Teager-Kaiser). For each treatment, pre- and post-exercise spectrograms depict 0.5-62.5Hz activity from -200 to 700ms for channels that demonstrated the largest evoked potentials and oscillatory activity: Fz and Cz. Difference spectrograms are plotted on the bottom axis. Spectrogram activity is interpolated and scaled against pre-exercise placebo values specific to each sensor. Full-spectrum activity is provided at the bottom of each panel, with pre- and post-exercise activity overlaid. ERP: event-related potential.

For the upper-body, at Fz, pre-exercise activity was concentrated from 250-450ms at theta frequency (**Fz**). After exercise, theta activity was reduced and distributed across an earlier latency range (125-250ms) for all treatments. Difference plots indicated a dose-dependent reduction in theta activity at 275ms, with 960mg retaining the most activity. At Cz, activity was concentrated from 150-200ms in the theta band (**Cz**).
