# Supplementary material for: Constitutive and Stress-Induced Psychomotor Cortical Responses to Compound K Supplementation
Source: Front Neurosci. 2020 Apr 8;14:315. doi: 10.3389/fnins.2020.00315 (PMC7158875; doi:10.3389/fnins.2020.00315)
Supplement: Supplementary file 4 [file Image_4.pdf]

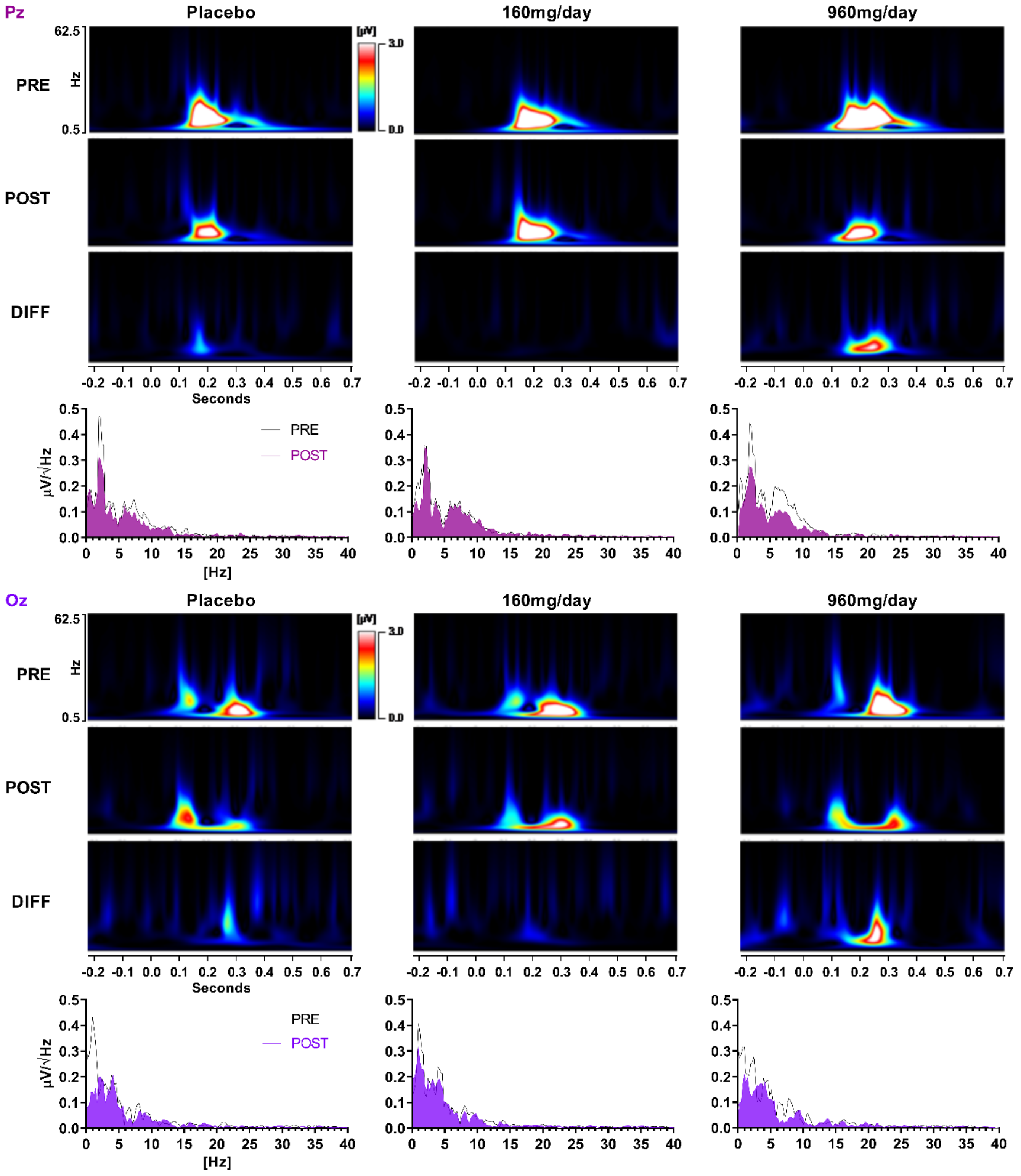

Supplementary Figure 4. Grand-Averaged Time-Frequency Spectrograms for Upper-Body Stimuli. Phase-locked spectral activity was examined with spectrograms of grand-averaged ERP waveforms using wavelet analysis (Mexican Hat, resolution = 1.0Hz, Teager-Kaiser). For each treatment, pre- and post-exercise spectrograms depict 0.5-62.5Hz activity from -200 to 700ms for channels that demonstrated the largest evoked potentials and oscillatory activity: Pz and Oz. Difference spectrograms are plotted on the bottom axis. Spectrogram activity is interpolated and scaled against pre-exercise placebo values specific to each sensor. Full-spectrum activity is provided at the bottom of each panel, with pre- and post-exercise activity overlaid. ERP: event-related potential.

At Pz, pre-exercise activity was concentrated from 100-300ms at alpha frequency and most pronounced for 960mg (**Pz**). At Oz, there was initial alpha activity from 100-160ms, followed by theta activity from 150-375ms (**Oz**). For placebo, post-exercise activity reductions were concentrated around 250-300ms at alpha/beta frequencies. For 160mg, there was little difference between timepoints. For 960mg, activity reductions were more broadly distributed from 150-300ms and concentrated at alpha frequency.
