# Supplementary material for: Constitutive and Stress-Induced Psychomotor Cortical Responses to Compound K Supplementation
Source: Front Neurosci. 2020 Apr 8;14:315. doi: 10.3389/fnins.2020.00315 (PMC7158875; doi:10.3389/fnins.2020.00315)
Supplement: Supplementary file 5 [file Image_5.pdf]

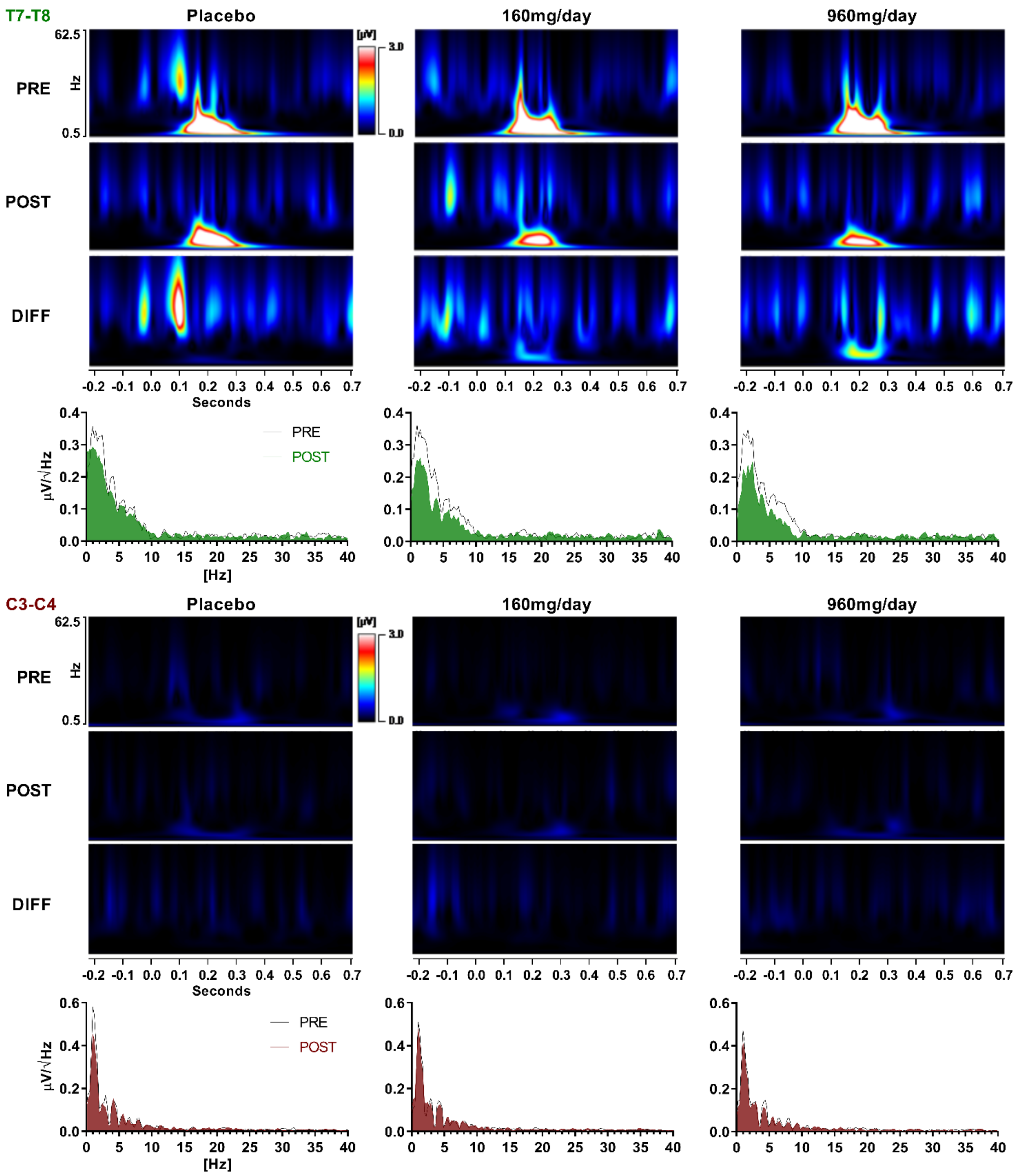

Supplementary Figure 5. Grand-Averaged Time-Frequency Spectrograms for Upper-Body Stimuli. Phase-locked spectral activity was examined with spectrograms of grand-averaged ERP waveforms using wavelet analysis (Mexican Hat, resolution = 1.0Hz, Teager-Kaiser). For each treatment, pre- and post-exercise spectrograms depict 0.5-62.5Hz activity from -200 to 700ms for channels that demonstrated the largest evoked potentials and oscillatory activity: T7-T8 average and C3-C4 average. Difference spectrograms are plotted on the bottom axis. Spectrogram activity is interpolated and scaled against pre-exercise placebo values specific to each sensor. Full-spectrum activity is provided at the bottom of each panel, with pre- and post-exercise activity overlaid. ERP: event-related potential

At temporal sites (**T7-T8**), pre-exercise activity was centered around 100-400ms for all treatments, with initial portions that contained more alpha and beta activity, and later portions with delta, theta, and alpha activity. After exercise, there was a dose-dependent reduction in activity. For placebo, earlier (-50-0ms and 75-125ms) clusters of beta and gamma activity were evident before exercise, but largely absent afterwards. From -100-0ms, active treatments had increased gamma activity after exercise. At lateral-central sites (**C3-C4**), pre-exercise activity was concentrated around 100-300ms with prominent alpha, beta, and gamma activity around 100ms and theta activity around 300ms. Activity in this range did not differ markedly between conditions or timepoints. Nevertheless, difference plots revealed a generalized reduction in beta and gamma activity after exercise.
