# Supplementary material for: Constitutive and Stress-Induced Psychomotor Cortical Responses to Compound K Supplementation
Source: Front Neurosci. 2020 Apr 8;14:315. doi: 10.3389/fnins.2020.00315 (PMC7158875; doi:10.3389/fnins.2020.00315)
Supplement: Supplementary file 6 [file Image_6.pdf]

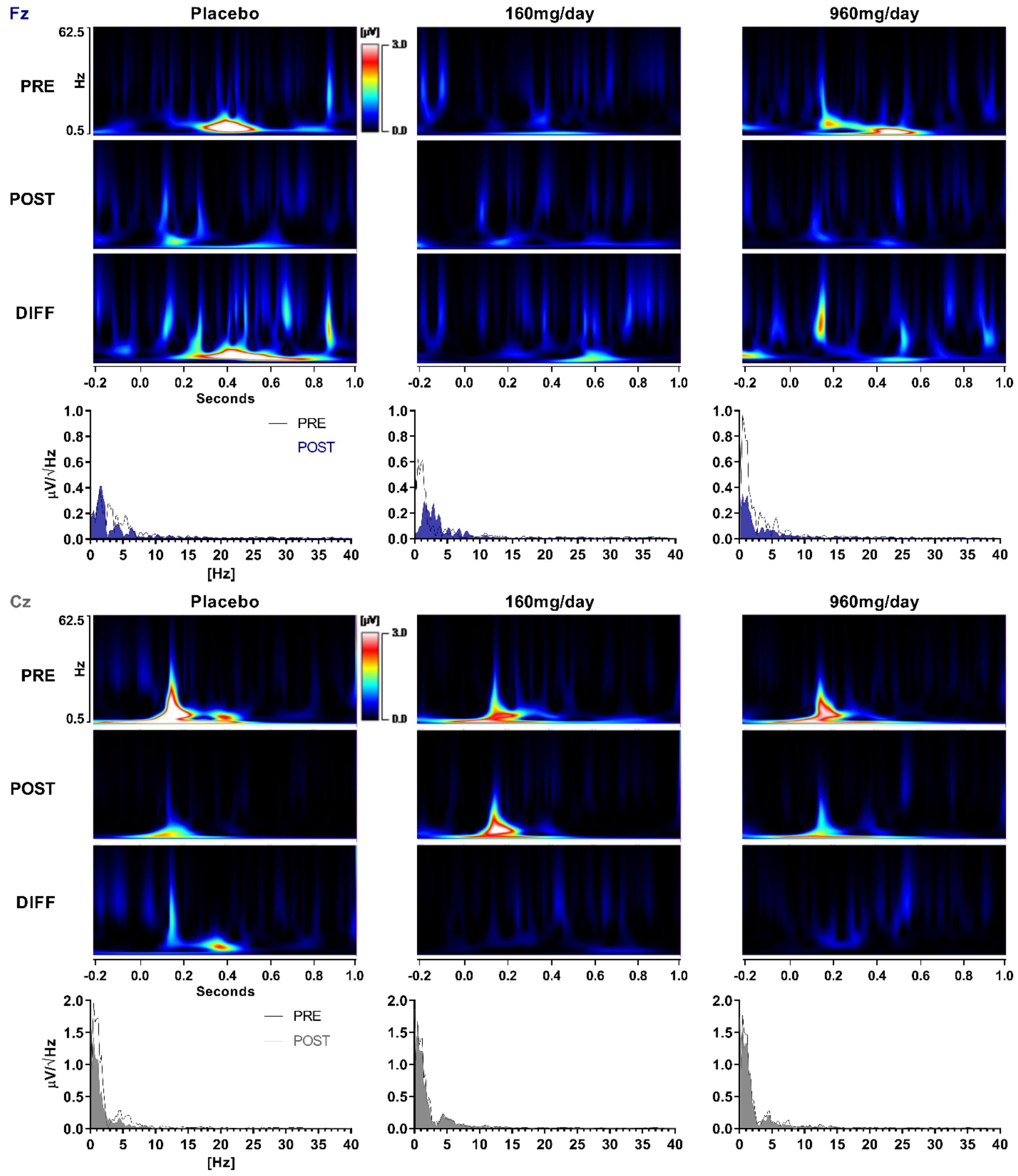

Supplementary Figure 6. Grand-Averaged Time-Frequency Spectrograms for Lower-Body Stimuli. Phase-locked spectral activity was examined with spectrograms of grand-averaged ERP waveforms using wavelet analysis (Mexican Hat, resolution = 1.0Hz, Teager-Kaiser). For each treatment, pre- and post-exercise spectrograms depict 0.5-62.5Hz activity from -200 to 1000ms for channels that demonstrated the largest evoked potentials and oscillatory activity: Fz and Cz. Difference spectrograms are plotted on the bottom axis. Spectrogram activity is interpolated and scaled against pre-exercise placebo values specific to each sensor. Full-spectrum activity is provided at the bottom of each panel, with pre- and post-exercise activity overlaid.

Lower-body CRTs had increased pre-exercise delta activity at Fz from 100-600ms (**Fz**). 960mg had distinct activity from 150-250ms at alpha/beta frequency, which was reduced after exercise. Placebo displayed greater reductions in theta activity after exercise, but delta activity was maintained. At Cz, pre-exercise activity was concentrated from -200-500ms at delta and theta frequencies (**Cz**). There was increased alpha and beta activity around 150ms, most notably for placebo. From pre- to post-exercise, all treatments had similar reductions in delta and theta activity, with additional alpha reductions for placebo.
