# Supplementary material for: Constitutive and Stress-Induced Psychomotor Cortical Responses to Compound K Supplementation
Source: Front Neurosci. 2020 Apr 8;14:315. doi: 10.3389/fnins.2020.00315 (PMC7158875; doi:10.3389/fnins.2020.00315)
Supplement: Supplementary file 7 [file Image_7.pdf]

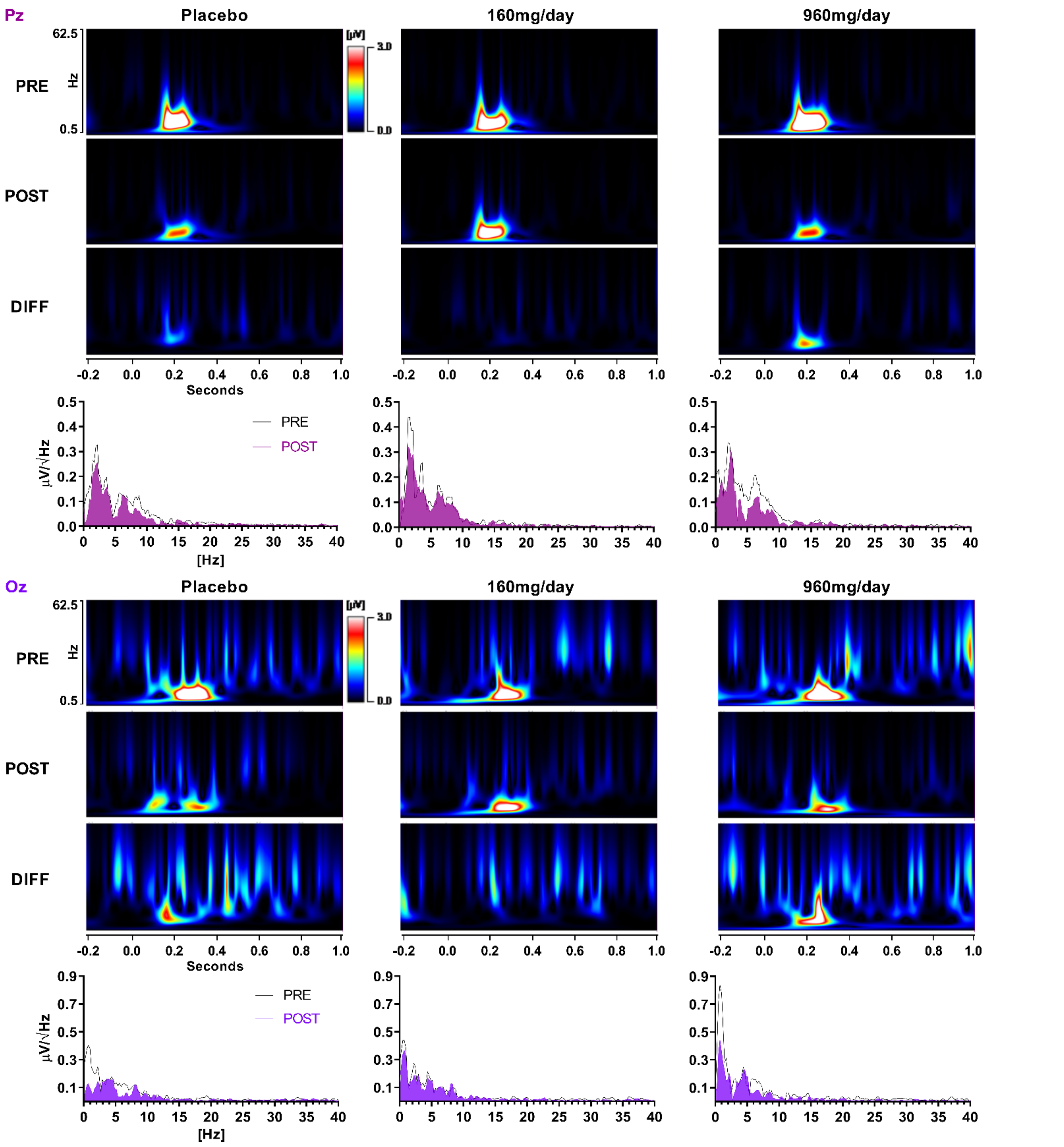

Supplementary Figure 7. Grand-Averaged Time-Frequency Spectrograms for Lower-Body Stimuli. Phase-locked spectral activity was examined with spectrograms of grand-averaged ERP waveforms using wavelet analysis (Mexican Hat, resolution = 1.0Hz, Teager-Kaiser). For each treatment, pre- and post-exercise spectrograms depict 0.5-62.5Hz activity from -200 to 1000ms for channels that demonstrated the largest evoked potentials and oscillatory activity: Pz and Oz. Difference spectrograms are plotted on the bottom axis. Spectrogram activity is interpolated and scaled against pre-exercise placebo values specific to each sensor. Full-spectrum activity is provided at the bottom of each panel, with pre- and post-exercise activity overlaid.

At Pz, pre-exercise activity was concentrated from 100-300ms at theta and alpha frequencies (**Pz**). Exercise reduced this activity for 960mg and placebo, while 160mg displayed similar activity at each timepoint. At Oz, pre-exercise activity was concentrated around 200-400ms at theta and alpha frequencies, with additional delta activity from 30-200ms (**Oz**). A post-exercise reduction in high frequency activity appeared throughout the analysis interval.
