# Supplementary material for: Constitutive and Stress-Induced Psychomotor Cortical Responses to Compound K Supplementation
Source: Front Neurosci. 2020 Apr 8;14:315. doi: 10.3389/fnins.2020.00315 (PMC7158875; doi:10.3389/fnins.2020.00315)
Supplement: Supplementary file 8 [file Image_8.pdf]

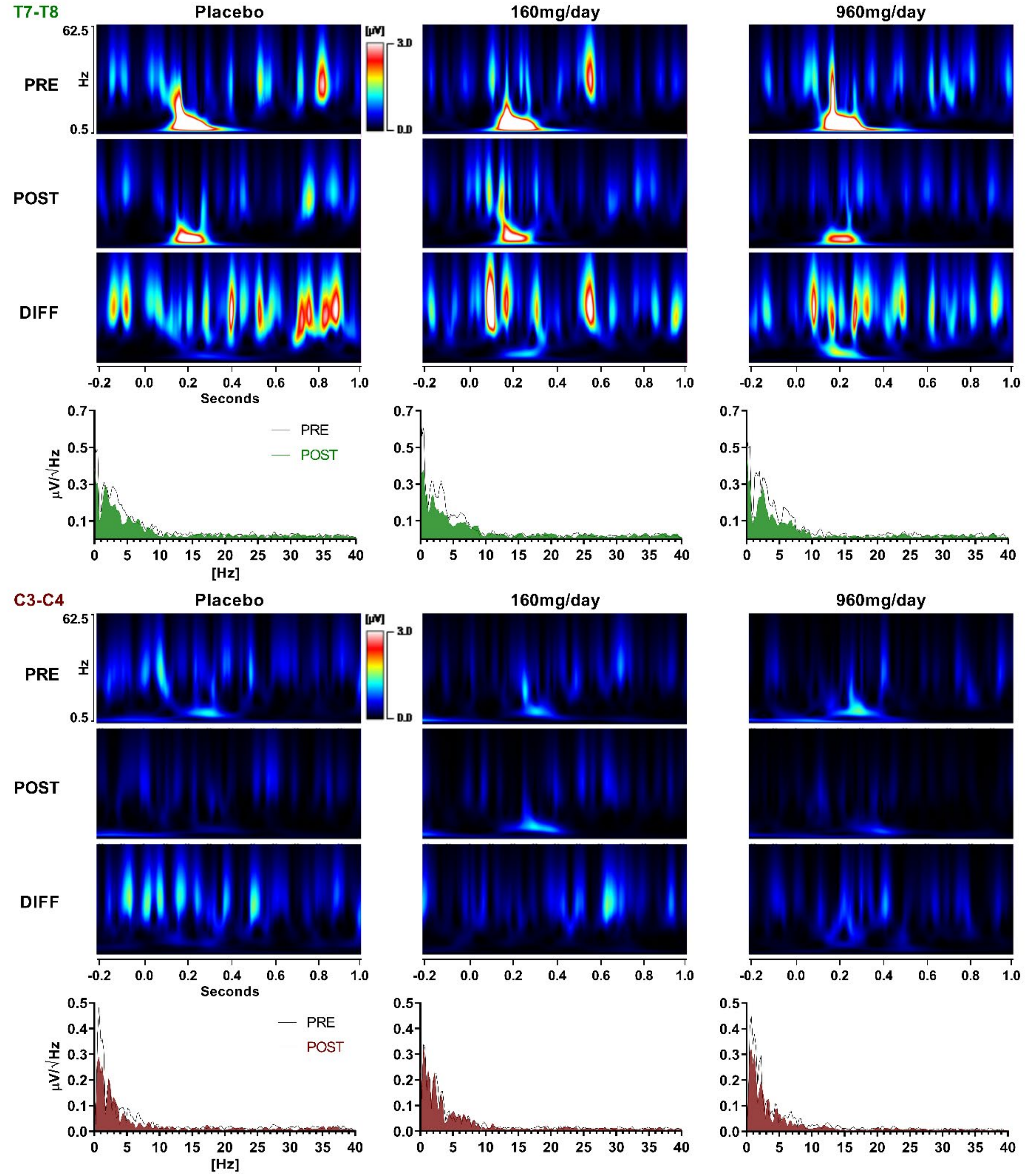

Supplementary Figure 8. Grand-Averaged Time-Frequency Spectrograms for Lower-Body Stimuli. Phase-locked spectral activity was examined with spectrograms of grand-averaged ERP waveforms using wavelet analysis (Mexican Hat, resolution = 1.0Hz, Teager-Kaiser). For each treatment, pre- and post-exercise spectrograms depict 0.5-62.5Hz activity from -200 to 1000ms for channels that demonstrated the largest evoked potentials and oscillatory activity: T7-T8 average and C3-C4 average. Difference spectrograms are plotted on the bottom axis. Spectrogram activity is interpolated and scaled against pre-exercise placebo values specific to each sensor. Full-spectrum activity is provided at the bottom of each panel, with pre- and post-exercise activity overlaid.

At temporal sites, pre-exercise theta activity was apparent from 115-250ms, with additional alpha/beta activity around 165ms for 960mg and placebo. After exercise, this activity decreased in a dose-dependent manner from 960mg to placebo (**T7-T8**). Placebo had less post-exercise beta and gamma activity from -200-100ms, 500-600ms, and 800-850ms, but increased activity around 735-785ms. The 160mg dose also had less beta and gamma activity from 500-600ms, whereas activity increased from 0-150ms and 640-870ms. 960mg displayed greater post-exercise reductions in beta and gamma activity generally, but especially over early (-200-100ms) and late (600-1000ms) intervals. At lateral-central sites (**C3-C4**), pre-exercise activity was concentrated from 250-350ms at theta/alpha frequency. After exercise, there was less theta and alpha activity for placebo and 960mg, while activity appeared similar for 160mg. A diffuse dose-dependent reduction in beta and gamma activity was apparent over the analysis interval from placebo to 960mg.
